# Assessing the Impact of Neuromuscular Electrical Stimulation-Based Fingerboard Training versus Conventional Fingerboard Training on Finger Flexor Endurance in Intermediate to Advanced Sports Climbers: A Randomized Controlled Study

**DOI:** 10.3390/s24134100

**Published:** 2024-06-24

**Authors:** Carlo Dindorf, Jonas Dully, Joshua Berger, Stephan Becker, Emanuel Wolf, Steven Simon, Eva Bartaguiz, Wolfgang Kemmler, Michael Fröhlich

**Affiliations:** 1 Department of Sports Science, University of Kaiserslautern-Landau (RPTU), 67663 Kaiserslautern, Germany; jonas.dully@rptu.de (J.D.); stephan.becker@rptu.de (S.B.); steven.simon@rptu.de (S.S.); eva.bartaguiz@rptu.de (E.B.); michael.froehlich@rptu.de (M.F.); 2 Department of Applied Training Science, German University for Prevention and Health Management, 66123 Saarbrücken, Germany; j-berger@dhfpg-bsa.de; 3 Institute of Medical Physics and Microtissue Engineering, Friedrich-Alexander-University of Erlangen-Nürnberg, 91054 Erlangen, Germany; wolfgang.kemmler@fau.de

**Keywords:** electromyostimulation, EMS, finger flexors, hang board, lead climbing, resistance training, sports, bouldering, training frequency, dead hang, neuromuscular adaptation

## Abstract

Competitive climbers engage in highly structured training regimens to achieve peak performance levels, with efficient time management as a critical aspect. Neuromuscular electrical stimulation (NMES) training can close the gap between time-efficient conditioning training and achieving optimal prerequisites for peak climbing-specific performances. Therefore, we examined potential neuromuscular adaptations resulting from the NMFES intervention by analyzing the efficacy of twice-weekly NMES-supported fingerboard (hang board) training compared with thrice-weekly conventional fingerboard training over 7 training weeks in enhancing climbing-specific endurance among intermediate to advanced climbers. Participants were randomly divided into the NMES and control groups. Eighteen participants completed the study (14 male, 4 female; mean age: 25.7 ± 5.3 years; mean climbing experience: 6.4 ± 3.4 years). Endurance was assessed by measuring the maximal time athletes could support their body weight (hanging to exhaustion) on a 20 mm-deep ledge at three intervals: pre-, in-between- (after 4 weeks of training), and post-training (after 7 weeks of training). The findings revealed that despite the lower training volume in the NMES group, no significant differences were observed between the NMES and control groups in climbing-specific endurance. Both groups exhibited notable improvements in endurance, particularly after the in-between test. Consequently, a twice-weekly NMES-supported fingerboard training regimen demonstrated non-inferiority to a thrice-weekly conventional training routine. Incorporating NMES into fingerboard workouts could offer time-saving benefits.

## 1. Introduction

The physical demands of climbing are influenced by various factors, including the discipline (bouldering, lead, speed), steepness of the route, climbing style, distance between holds, hold dimensions, and overall challenge of the climb [1,2,3,4,5,6]. A growing body of scientific research has investigated the physiological requirements of climbing and explored the connection between climbing performance, muscular strength, and endurance [6]. Various physiological elements, including flexibility [7,8], finger strength [9,10], and endurance [11] have been shown to be indicators of climbing performance and can be used to assess climbing performance. Grip strength and endurance are pivotal determinants of climbing proficiency [12,13,14], besides factors like psychological parameters [15,16]. For instance, a previous study revealed that elite climbers (8b–8c Fontaine scale redpoint) exhibited higher levels of finger strength and arm endurance compared with those of advanced climbers (7c + −8a Fontaine scale redpoint) [17].

Climbing training often involves activities closely resembling competition scenarios, such as bouldering sessions on climbing walls [18] or grip-specific strength and endurance training [19]. Existing studies underscore the positive impact of fingerboard training on climbing-specific performance [20,21,22]. A meta-analysis highlighted that resistance training enhanced performance in climbing-specific assessments, such as dead-hang duration or finger strength, compared with standard climbing training [18].

Competitive climbers engage in highly structured training regimens to achieve peak performance levels [23]. Efficient time management is critical at this elite level, necessitating the integration of diverse training elements within the limitations of available time and physiological feasibility. Implementing time-saving strategies, such as enhancing the efficiency of fingerboard training, could yield benefits by providing additional time for other training components or enabling extended recovery periods [19].

One potential method to address this challenge is neuromuscular electrical stimulation (NMES) training [24]. During electrical muscular stimulation (EMS), an electrical impulse is applied to the muscle via electrodes attached to the skin, triggering an involuntary contraction of the underlying muscles. A distinction is made between the local application of NMES with one to five pairs of electrodes and whole-body NMES (WB-NMES), which involves the use of at least six pairs of electrodes targeting all major muscle groups, delivering a training-effective impulse that induces muscular adaptations [25,26]. The two types of application differ only in the number of electrodes used; the stimulation parameters and the application sites can be selected identically. Research across various sports has demonstrated performance enhancements with local and WB-NMES [26,27], depending on the specific movement or task. Improved performance of the knee extensors and flexors, the hip adductors, and the trunk flexors was observed after a 10-week WB-NMES training intervention, which was carried out as a substitute for conventional athletic training for young soccer players [26], indicating the time efficiency of WB-NMES in significantly enhancing performance. In badminton players, a significant improvement in standing broad jump performance and vertical jump height has also been observed with local EMS application over both lower limbs’ quadriceps femoris and triceps surae muscles over a 4-week period [28]. After a 12-week EMS application of the knee extensors, plantar flexors, and gluteus muscles, rugby players benefited from increased isokinetic maximum strength in eccentric (18.0 ± 26.3%) and concentric (19.4 ± 28.9%) conditions, increased 1RM squat strength (15.0 ± 8.0%), and the jump height of the squat jump (10.0 ± 9.5%) and drop jump from a 40 cm height (6.6 ± 6.1%) also increased significantly [29]. In past studies, athletes from other sports, such as ice hockey, cycling, and basketball, have also benefited from both WB-NMES and the local application [24,30]. Physiologically, this could be due to the fact that GK-EMS training appears to result in a reversal of the recruitment pattern so that faster type II fibers are stimulated at an early stage. This occurs because larger motor units have larger axons, which have a lower stimulation threshold than smaller axons, which occur in small motor units, and these larger motor units are often close to the skin surface, which may favor early stimulation. Studies speak of a non-selective, synchronous recruitment of the motor units during EMS training, which implies a simultaneous stimulation of both muscle fiber types at low force production and positively causes training of the faster FT fibers even in low intensity ranges [31,32,33].

Despite these promising findings, the application of NMES in climbing remains relatively unexplored. To the best of the authors’ knowledge, only one study has focused on NMES training in climbing [34]. This study demonstrated significantly improved isokinetic wrist strength and endurance using NMES-supported fingerboard training compared with the strength and endurance observed with conventional fingerboard training. However, the participants in that study were inexperienced climbers, who may have a greater potential for improvement compared with intermediate and advanced climbers. Consequently, significant research gaps persist in this domain.

The efficiency of NMES in achieving muscular adaptations with reduced training time compared with other forms of training or performing the exercise content alone without additional NMES has already been clearly demonstrated [26], which is why climbing athletes could also benefit from the effects of additional NMES. Building on this, it is conceivable that incorporating NMES into fingerboard training may reduce the frequency of training sessions compared with conventional methods. Thus, based on the outlined research gap, we aimed to examine whether a twice-weekly NMES-supported fingerboard training regimen, despite its reduced training volume, can demonstrate non-inferiority to a three-weekly conventional training routine concerning the endurance improvements of the finger flexor muscles of intermediate to advanced climbers.

## 2. Materials and Methods

### 2.1. Study Overview

**Figure 1:**
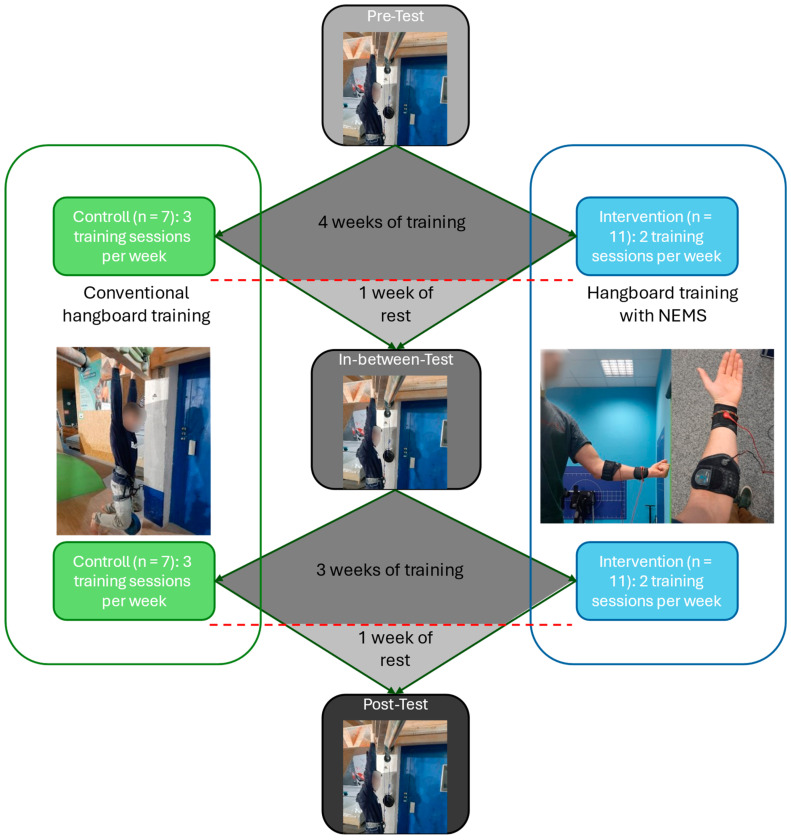
Workflow of the data acquisition and the different training setups (NMES = neuromuscular electrical stimulation).

The present NMES randomized controlled trial (RCT) was conducted in a parallel group design and initiated in June 2023. In this study, the term “control group” refers to the group that received no NEMS application, while the term “treatment group” refers to the group that received an NEMS application. The trial was planned, initiated, and conducted by the Department of Sports Science, University of Kaiserslautern–Landau, Germany. The Ethics Committee of the University of Kaiserslautern–Landau provided official consent (ID: 58; date of approval: 27 April 2023) in complete adherence with the Helsinki Declaration “ethical principles for medical research involving human subjects” [35]. The workflow of data acquisition and the different training setups are presented in Figure 1 and described in more detail below. For an overview of the primary and secondary variables used in the study, which are described in more detail below, please refer to Table A1 in Appendix A.

### 2.2. Participants

Participants were required to be at least 18 years old and have at least 2 years of active climbing experience with a boulder grade of at least 6b (Fontaine scale). The Fontaine scale, also referred to as the Fontainebleau or French scale, ranges from 4 to 9a [36]. The exclusion criteria included acute injuries or complaints associated with fingers, arms, shoulders, or trunks, as well as contraindications for conventional usage of NMES [37,38]. Participants were recruited based on personal contact with the athletes and coaches, as well as through additional flyers posted in the bouldering gym. After providing comprehensive information to all study participants, written informed consent was obtained from each of them.

Initially, 22 athletes were recruited and randomly assigned to either the NMES group or the control group using a computer-generated randomization sequence to ensure unbiased allocation. Of these participants, 18 athletes (14 male and 4 female; age: 25.7 ± 5.3 years) completed the study. Four participants dropped out of the control group, while the NMES group reported no dropouts, resulting in seven and 11 participants in the control and NMES groups, respectively (refer to the CONSORT flowchart in Appendix A). 

Participants self-reported their years of climbing experience, weekly time devoted to each discipline, and climbing ability levels in both bouldering and lead climbing on the International Rock Climbing Research Association (IRCRA) scale. The scale ranges from 1 to 33 and promises greater comparability and better usage in statistical analysis [39]. Body weight was measured at pre-, in-between, and post-test time points using a digital scale (OMRON BF511, Omron Healthcare Co., Ltd., Mannheim, Germany). Corresponding detailed participant characteristics for each group are presented in Table 1.

### 2.3. Assessment Outcomes

The study’s assessment outcomes are categorized as follows: 

Primary outcomes:

- Interaction effect of group (control, NMES) and performance assessment time point (pre, in-between, post) for finger flexor endurance.

Secondary outcomes:

- Changes in finger flexor endurance from a baseline (pre-test) to a 4-week assessment (in-between).
- Changes in finger flexor endurance from a baseline (pre-test) to a 7-week assessment (post-test).
- Changes in finger flexor endurance from a 4-week assessment (in-between) to a 7-week assessment (post-test).

### 2.4. Performance Testing

Endurance assessments were conducted at three different points (pre, in-between, and post). Following the pre-test, participants trained for 4 weeks, with the in-between test conducted during the fifth week. Subsequently, participants resumed training for an additional 3 weeks before undergoing the post-test. A standardized one-week rest period preceded both the in-between and post-tests, consistent with that in previous NMES interventions [40]. Our choice of one-week rest aligns with previous research using a similar rest period (roughly one week) before final testing following fingerboard training [41]. While one study reported improved finger endurance after 48 h of rest [22], a one-week rest period ensures a more complete recovery, minimizing the potential for testing to be influenced by fatigue. Consequently, the rest duration ensures adequate recovery and allows sufficient time for training adaptations to occur. This selected rest period also adheres to common training routines used by climbers and avoids disrupting their weekly planned training schedules. 

Endurance was assessed by measuring the maximal time athletes could support their body weight (hanging to exhaustion) in two attempts, following the methodology outlined by a previous study, as it has been shown to correlate with climbers’ competence [17].

**Figure 2:**
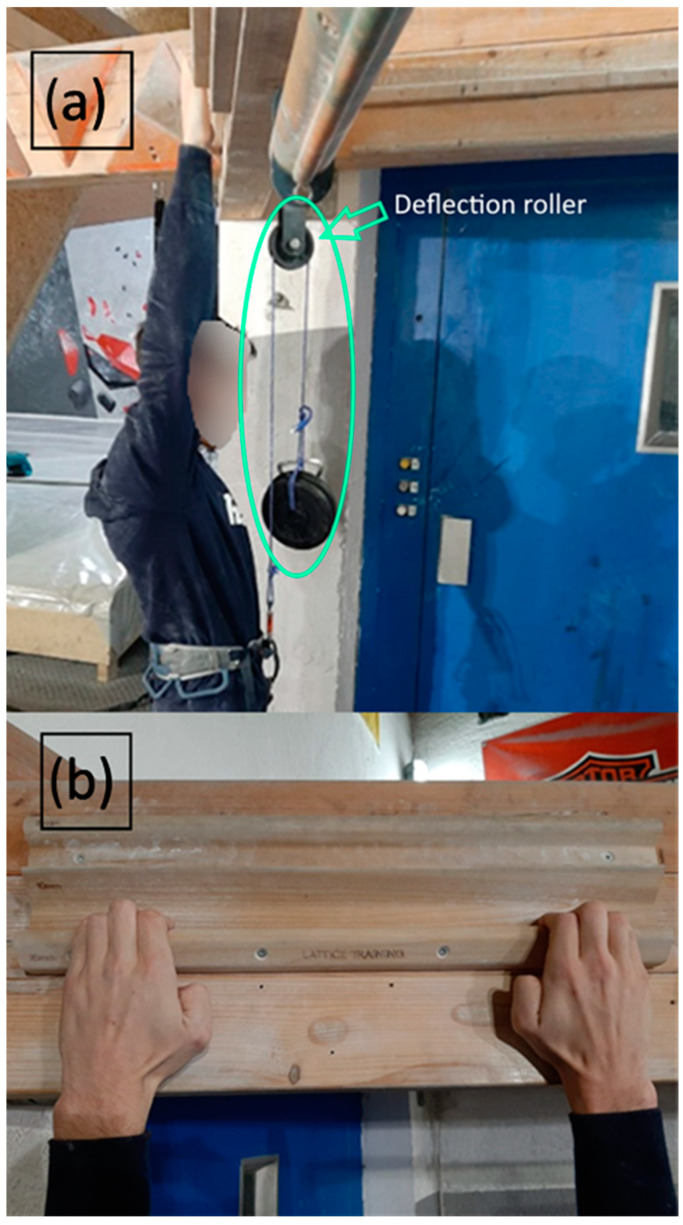
(**a**) Setup for performance testing and training on the fingerboard, including weight supported by a deflection roller. (**b**) A 20 mm-deep ledge with 10 mm of rounding was used in the study.

For the determination of the adaptation of the training intensity of the intervention, maximal strength was used as a reference and measured to be adapted to the lattice training [42]. Maximal strength was defined as the weight (expressed as a percentage of body weight) athletes could sustain for (body weight minus counterweight used for assistance or body weight plus additional weight if the climber can hang on the hold) 7 s on the specified ledge [43]. Recognizing that the maximal strength might be lower than the athlete’s own body weight, the weight was supported by a deflection roller (Figure 2). If a participant could not maintain the position for 7 s, the attempt ended. In the subsequent trial, the additional load was reduced by 1–2 kg to establish the individual maximum load they could sustain. 

Between individual trials, participants were given 3 min of recovery time, which, according to [44,45,46], is considered sufficient for complete recovery. A 20 mm-deep ledge with 10 mm of rounding (Lattice Training Rung, Lattice Training Ltd., Chesterfield, UK) was utilized for testing endurance and strength. Participants were instructed to refrain from hard physical activity for 48 h before testing. Tests and training sessions were conducted with straight arms, stabilized shoulders, and cores and were terminated, if athletes assumed a non-stabilized position. For the half-crimp position, four fingers (excluding the thumb) were placed on the ledge with the flat side of the fingertips in contact with the surface. 

### 2.5. Intervention

The athletes were allowed to continue their regular climbing routine during the investigation. Each training session of the intervention consisted of a standardized 15 min warm-up comprising low-intensity climbing, kneeling push-ups, jumping jacks, shoulder shrugs, and no-hang grips (hanging with counteracting part of the body weight without lifting feet off the ground) on the board. The training comprised six identical sets, interspersed with 2 min breaks between each set. Each set comprised six repetitions of hanging with straight arms and stabilized shoulders for 7 s, followed by 3 s breaks on the designated ledge used for performance assessments. The break duration and the 7:3 split were determined based on guidance from experienced climbing trainers of the participants, familiarity with the protocol due to previous experience, and insights from current literature [22], except for an increased number of sets to accommodate the participants’ training levels. The training was planned to nearly induce muscle failure, with termination occurring when repetitions within a set could not be fully completed for more than three consecutive attempts.

For both groups, each training session was conducted with 70% of the individual maximal strength measured or adjusted, respectively, during the pre- and in-between tests as described above (see Section 2.4). This intensity level was selected based on reported positive outcomes for finger flexor endurance [47]. For this purpose, participants were either provided with additional weight or aided by a weight-support mechanism using a deflection roller (refer to Figure 2).

### 2.6. Neuromuscular Electrical Stimulation Setup

**Figure 3:**
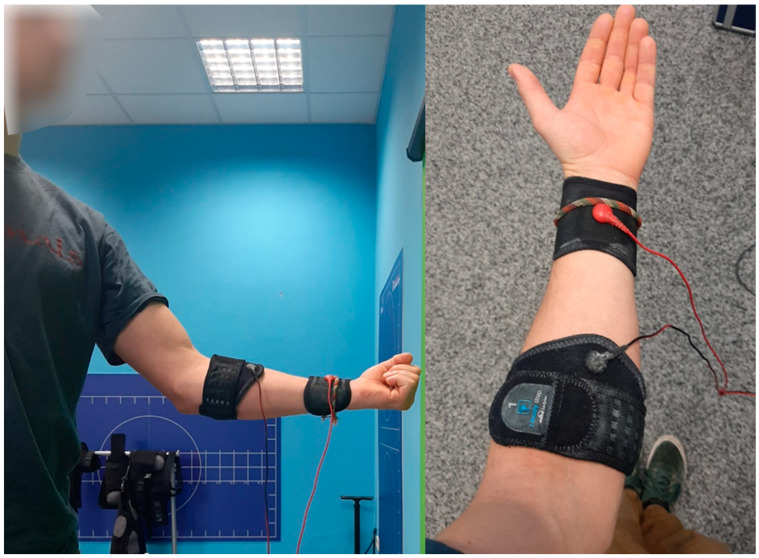
Placement of the electrical muscular stimulation electrodes on the proximal and distal parts of the finger flexor muscles.

For the participants in the NMES Group, the distal and proximal parts of the finger flexors were stimulated with an NMES device (Miha Bodytec GmbH, Gersthofen, Germany) applied to the forearm (Figure 3), to which the athletes were habituated before the training sessions. Impulse intensity increased during habituation and training to prevent habituation to one specific activation, as a previous study had shown that the maximum tolerable stimulus intensity adapts successively in successive units [48]. The intensity was consistently adjusted to align with the subjective sensation, ensuring it remained above the level of involuntary muscle contraction, as assessed by the CR-10 Borg scale [49], with a targeted Borg scale value of 6 [26].

The electrical impulses were delivered only for 5 out of the 7 s under load, allowing a stress-free transition into the hanging position. A frequency of 85 Hz with an impulse width of 350 ms and a rise time of 0.4 s was selected according to a previous study [50]. The use of these stimulation parameters has become established in the WB-EMS application for effective performance enhancement, which is why they are also used in the local application carried out in this study. Directly after each training intervention, participants rated their subjective perceived total training exertion on the Borg 6–20 RPE scale [51] to enable a comparison between the perceived exertion of the training sessions for the NMES and control groups. The scale is commonly used in climbing studies [52,53,54] and has proven to be a good indicator of the physiological demands of advanced climbers [55]. The use of the two different Borg scales is due to the participants’ familiarity with indicating intensities using the CR-10 Borg scale and assessing total effort after a workout with the Borg 6–20 scale during regular training. We chose not to change this practice to leverage their existing experience with these scales.

### 2.7. Statistical Analysis

Single-tailed tests were conducted to determine whether a twice-weekly NMES-supported fingerboard training regimen could demonstrate non-inferiority to a thrice-weekly conventional training routine with regard to the endurance (and strength, as a control for our treatment) of the finger flexor muscles. Owing to non-normally distributed variances, a mixed robust analysis of variance was calculated using the WRS2 package [56] in R (version 4.4.0, R Software for Statistical Computing, Vienna, Austria) [57] with interaction effects of time of measurement (pre, in-between, post) and group (EMS training, conventional training). We opted for a trimmed means approach of 10% since it consistently produced results similar to those of the 20% trimming method while exerting less influence on the measurement data. The *p*-values were adjusted using the Bonferroni correction for post-hoc testing. Potential differences in participants’ climbing or bouldering ability level (self-rated lead climbing and bouldering ability level on the IRCRA scale) between the NMES and control groups were analyzed using *t*-tests. The Spearman correlation was utilized to explore the relationship between participants’ climbing or bouldering ability level and endurance changes, given the non-normal distribution. The level of significance was set at *p* = 0.05. Visualizations were performed using the Python libraries Seaborn [58] and MatPlotLib [59].

## 3. Results

### 3.1. Primary and Secondary Outcomes

Table 2 shows the intra- and intergroup results. Mixed robust analysis of variance showed no statistically significant interaction between time and group for endurance (*F*(2, 6.76) = 0.88, *p* = 0.23). According to Cohen [60], a strong main effect for time was observed (*F*(2, 6.76) = 8.43, *p* = 0.02, partial *η*^2^ = 0.71, *f* = 1.58) but not for the group (*F*(1, 4.49) = 0.04, *p* = 0.86) regarding endurance.

**Figure 4:**
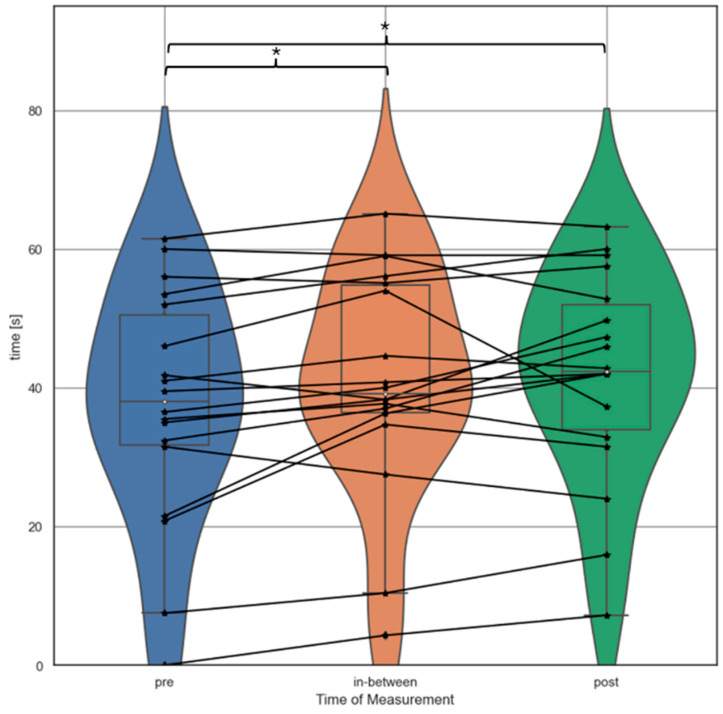
Violin plots illustrating the results of the endurance test (time athletes could support their body weight; hanging to exhaustion) at the three points of measurement: pre, in-between, and post. Black lines represent individual changes. Within each violin, box-and-whisker plots are displayed, with the white inner line representing the median value. * indicates significant differences.

Post-hoc analysis demonstrated differences between the pre- and in-between tests (*t* = −3.17, *p* = 0.01, *n* = 18, *r* = 0.61), as well as between the pre- and post-tests (*t* = −2.60, *p* = 0.02, *n* = 18, *r* = 0.53), both with a strong effect size, whereby an increase in endurance was noted. Individual holding time increased on average by 3.68 ± 4.93 s between the pre- and in-between tests and 4.51 ± 7.36 s between the pre- and post-tests. Excluding the single subject, which could not support his body weight at pre-test time of measurement, this represents a mean individual improvement of 14.30 ± 23.01% from pre- to in-between test and 20.45 ± 36.94% pre- to post-test. No statistical difference was identified between the in-between- and post-tests (*t* = −0.53, *p* = 0.60, *n* = 18; Figure 4).

### 3.2. Additional Results

No differences between NMES and the control group exist for the self-rated ability level (climbing: *t*(16) = 1.19, *p* = 0.25; bouldering: *t*(7.85) = 0.92, *p* = 0.38), as determined by *t*-tests. On average, participants assessed their perceived exertion of the training sessions using the Borg 6–20 scale, yielding ratings of 15.0 ± 2.4 and 14.6 ± 2.8 points for the NMES and control groups, respectively. The participants in the NMES group achieved an average completion rate of 72.0 ± 20.0%, while those in the control group completed 96.0 ± 9.0% of the planned training sets until termination according to the specified criteria. 

No main effects for both time (*F*(1, 6.45) = 0.84, *p* < 0.47) and group (*F*(1, 4.38) = 0.15, *p* = 0.72) were identified for maximal strength according to the mixed robust analysis of variance. Spearman correlation showed no correlation between participants’ climbing and bouldering ability level and endurance changes between pre- and in-between tests (climbing: *r* = 0.04, *p* = 0.86, *n* = 18; bouldering: *r* = −0.25, *p* = 0.32, *n* = 18) as well as pre- and post-tests (climbing: *r* = −0.22, *p* = 0.39, *n* = 18; bouldering: *r* = −0.47, *p* = 0.05, *n* = 18).

## 4. Discussion

The results of this study revealed that a twice-weekly NMES-supported fingerboard training regimen demonstrated non-inferiority to a thrice-weekly conventional training routine in terms of effects on finger flexor endurance. Comparable improvements between twice-weekly NMES training and thrice-weekly conventional training were observed. Therefore, the incorporation of NMES training into fingerboard workouts exhibits the potential for enhancing endurance training efficiency while simultaneously reducing overall training frequency and volume. Notably, the most significant effects were observed after 4 weeks (in-between tests). However, continuing training for an additional 3 weeks (post-test) surprisingly did not yield further endurance improvements compared with those experienced in the in-between test, suggesting a potential plateau effect or diminishing returns with prolonged training. This observation resonates with the findings from another study on top world-ranking climbers, where a 4-week fingerboard intervention notably improved unilateral finger flexor rate of force development compared with that with regular climbing training, underscoring the possibility of diminishing returns with extended training efforts [5].

Individual holding time increased on average 4.51 ± 7.36 s between the pre- and post-tests, which makes an individual improvement of 20.45 ± 36.94% of the baseline value. The high standard deviation, as well as the data illustrated in Figure 4, indicate significant variation in endurance effectiveness among participants. The results revealed no correlation between participants’ climbing and bouldering ability levels and changes in endurance between the pre-test and in-between test or between the pre-test and post-test. Thus, the observed high variation in endurance effectiveness does not appear to be related to climbing ability level. Consistent with our findings, ref. [61] demonstrated a mean enhancement of 5.4–6.7 s in maximal dead hang time across various grips after four weeks of fingerboard training, involving three standardized sessions per week. Similarly, significant improvements from 10 weeks of twice-weekly fingerboard training alongside regular climbing training in maximal dead hang time of an average of 6.8 s have been discovered [22].

The lone study that delved into NMES finger training in climbing specifically targeted inexperienced climbers [34]. However, prior research suggested that in inexperienced climbers with low performance levels, enhancements in forearm endurance might be attributed to non-specific training methods, such as those utilizing dumbbells [18,22]. Consequently, the applicability of these findings may not extend to intermediate to advanced climbers, who exhibit distinct adaptations to training stimuli. Therefore, our study expanded the scope of research and demonstrated that for intermediate to advanced climbers, NMES training could yield effects on endurance as measured by dead hang time.

Maximum strength is an important contributing factor for local muscle endurance performance because strength endurance can be positively influenced by strength endurance-specific low-load, high-repetition resistance training as well as resistance training modalities that lead to an increase in maximum strength. NMES is an effective means for improving maximum strength and therefore bears the potential to improve muscle health as well as functional and clinical outcomes directly and indirectly through an increase in strength endurance performance [62,63]. However, in the current study, no significant effects on maximal strength were observed over time or between groups. This lack of observed effect might be due to the selected intensity level of 70% of individual maximal strength, which is known to produce positive outcomes for finger flexor endurance rather than maximal strength [47]. Consequently, the intensity may have been insufficient in this context to elicit changes in maximal strength.

Future studies can use WB-NMES training in climbing to evaluate the effects on overall climbing performance, as this can lead to improved functionality of the trunk muscles [26,64]. Consequently, this leads to an increasingly stable and controlled movement [64]. Previous studies have demonstrated significant increases in the maximum strength of the trunk and lower extremities in football players [26], cyclists [27], and untrained participants [65], suggesting that climbers could also benefit from WB-NMES in terms of general muscular performance of the trunk and lower extremities. Detached from this general capacity, the realization of a superimposed WB-NMES could be beneficial, as climbing-specific movement patterns are combined with an involuntary movement, which can lead to performance improvements in the sport-specific target movement [66,67]. Nevertheless, local NMES training still presents theoretical opportunities. Especially, the electrical stimulus has the potential to induce neuronal adaptations of the muscles, such as frequency and recruitment synchronization. These subtle adaptations may confer significant advantages for athletes, so that small intramuscular adaptations of the muscle fibers (frequency, recruitment, synchronization) already lead to decisive advantages for the athletes [33,68]. However, to date, this remains a hypothesis that has yet to be confirmed.

Training protocols for hang board exercises regulate intensity through either increased weight or smaller holds [20]. According to [20], increasing weight is the more effective approach. Hence, this method of intensity modulation was selected for the current study. A previous study determined that maximal dead hang times from a 25 mm ledge (R = 0.54) and a bar (R = 0.56) were highly correlated with climbers’ competence regarding muscle endurance [17]. However, considering the corresponding findings, slight adaptations were made for the present study. A 20 mm-deep ledge with 10 mm of rounding was utilized for testing endurance and strength instead of a 25 mm ledge. This adjustment was made based on participants’ familiarity with the 20 mm ledge, which stemmed from their training history.

This study had several limitations. First, the relatively modest sample size raises questions concerning the generalizability of the results. Hence, caution is warranted when extrapolating these findings, particularly to broader populations not represented in the study, such as less experienced athletes. Additionally, the participants exhibited a relatively heterogeneous ability level. Notably, no discernible climbing and bouldering ability differences were detected between the NMES group and the control group, suggesting an absence of systematic bias attributable to varying performance levels. Nevertheless, the heterogeneous nature of participant ability warrants consideration when analyzing the outcomes, notwithstanding the absence of observed correlations between individual ability levels and the intervention’s effects. Furthermore, highlighting that in this study, dead hang time served as the primary metric for assessing endurance, known to correlate significantly with climbing competence, is imperative [17]. However, endurance evaluated by submaximal, intermittent contractions of the finger flexors is reportedly associated with climbing ability [11]. Hence, future research endeavors should incorporate an evaluation of this aspect alongside the measures utilized in this study to provide a comprehensive understanding of climbing performance and endurance.

## 5. Conclusions

The results of the present study show that the twice-weekly NMES-supported fingerboard training regimen demonstrates non-inferiority to the thrice-weekly conventional training routine in improving finger flexor endurance. Therefore, the integration of NMES training into fingerboard workouts holds promise for optimizing endurance training efficiency and reducing the overall training frequency and volume. This potential could translate into improved athletic performance and enhanced recovery, capitalizing on the additional time made available by the proposed NMES-based approach.

## Figures and Tables

**Table 1:** Participant characteristics in each group and total.

	NMES Group *n* = 11		Control Group *n* = 7	
	Male *n* = 9	Female *n* = 2	Male *n* = 5	Female *n* = 2
Weight pre [kg]	71.6 ± 6.4	54.5 ± 6.4	79.0 ± 6.1	54.5 ± 3.5
Weight in-between [kg]	72.2 ± 6.4	55.0 ± 7.1	80.0 ± 5.0	54.0 ± 4.2
Weight post [kg]	72.7 ± 6.6	55.0 ± 7.1	80.0 ± 5.6	54.5 ± 5.0
Height [cm]	182.9 ± 5.0	162.5 ± 3.5	181.0 ± 2.0	163.0 ± 4.2
Age [years]	25.9 ± 5.5		25.4 ± 6.1	
Years of bouldering or climbing experience [years]	5.6 ± 3.1		7.6 ± 3.9	
Weekly time devotion per discipline [min]	Bouldering: 354.1 ± 64.8 Lead: 81.6 ± 109.2		Bouldering: 420.0 ± 120.0 Lead: 77.4 ± 88.8	
Lead climbing ability level [IRCRA scale]	16.36 ± 3.35		18.29 ± 3.35	
Bouldering ability level [IRCRA scale]	19.64 ± 1.96		21.14 ± 4.02	

**Table 2:** Descriptive intra- and intergroup results. “Merged” represents the descriptive values resulting from the combination of the neuromuscular electrical stimulation (NMES) and control groups.

Group	Pre	In-Between	Post
NMES [s]	37.47 ± 12.13	42.50 ± 11.10	42.55 ± 11.63
Control [s]	37.11 ± 23.95	38.69 ± 22.79	40.71 ± 20.78
Merged [s]	37.33 ± 17.00	41.02 ± 16.11	41.84 ± 15.26

**Figure A1:**
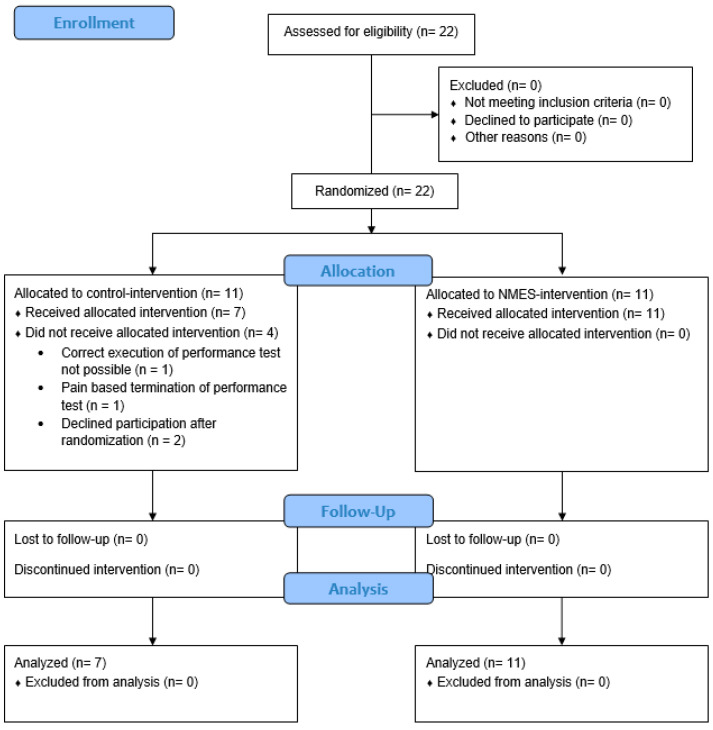
CONSORT flowchart of the study.

## Data Availability

The dataset for this article is available. Requests to access the datasets should be directed to the corresponding author.

## Appendix A

**Table A1:** Overview of the primary (main variable of interest) and secondary variables (used for descriptive purpose, exploration or to adjust treatment) used in the study.

Variable	Type	Description
endurance	primary	maximal time athletes could support their body weight (hanging to exhaustion)
maximal strength	secondary	weight (expressed as a percentage of body weight) athletes could sustain for (body weight minus counterweight used for assistance or body weight plus additional weight if the climber can hang on the hold) 7 s on the specified ledge
weight	secondary	body weight at pre-, in-between, and post-test time points
age	secondary	age of participants in years at the time of the pre-test
height	secondary	body height
previous experience	secondary	year of previous climbing or bouldering experience
weekly time deviation bouldering	secondary	self-reported weekly training time in bouldering
weekly time deviation lead climbing	secondary	self-reported weekly training time in lead climbing
lead climbing ability level	secondary	self-rated lead climbing ability level reported on IRCRA scale
bouldering ability level	secondary	self-rated bouldering ability level reported on IRCRA scale
perceived training exertion	secondary	participants rating of their subjective perceived total training exertion on the Borg 6–20 RPE after each training
training sets performed	secondary	training sets performed till termination according to termination criteria
